# West Nile Virus Seroprevalence, Connecticut, USA, 2000–2014 

**DOI:** 10.3201/eid2304.161669

**Published:** 2017-04

**Authors:** Megan E. Cahill, Yi Yao, David Nock, Philip M. Armstrong, Theodore G. Andreadis, Maria A. Diuk-Wasser, Ruth R. Montgomery

**Affiliations:** Yale University School of Public Health, New Haven, Connecticut, USA (M.E. Cahill);; Yale University School of Medicine, New Haven (Y. Yao, D. Nock, R.R. Montgomery);; The Connecticut Agricultural Experiment Station, New Haven (P.M. Armstrong, T.G. Andreadis);; Columbia University, New York, New York, USA (M.A. Diuk-Wasser)

**Keywords:** West Nile virus, aging, immune response, seroconversion, viral susceptibility, viruses, Connecticut, United States, vector-borne infections

## Abstract

West Nile virus (WNV) infection is mainly asymptomatic but can be severe in elderly persons. As part of studies on immunity and aging in Connecticut, USA, we detected WNV seroconversion in 8.5% of nonimmunosuppressed and 16.8% of immunosuppressed persons. Age was not a significant seroconversion factor. Our findings suggest that immune factors affect seroconversion.

 Since the 1999 emergence of West Nile virus (WNV) in North America, >43,000 cases of disease and 1,884 deaths have been reported (*1*); overall infections are estimated at ≈3 million (*2*). Although WNV infections can be asymptomatic, they can also cause severe neuroinvasive disease, especially among infants, immunocompromised persons, and elderly persons (*3*). Control of WNV infection involves innate immune pathways that mediate initial recognition and regulation of viral replication and adaptive immune responses that provide long-term protection (*3*). Spatial distribution analysis and mosquito surveillance studies have confirmed that WNV is endemic to Connecticut, USA (*1*,*4*). 

We compared seroprevalence and demographics for 890 nonimmunosuppressed and 173 immunosuppressed adults enrolled in a study on immunity in aging (approved by the Human Investigations Committee of Yale University) (*5*) with those of symptomatic WNV case-patients reported to the Connecticut Department of Health (DPH) during 2000–2014. DPH-reported symptomatic case-patients (n = 116) sought medical attention and had a positive WNV laboratory test result (*1*). None of the asymptomatic participants were reported to DPH as WNV case-patients. Immunosuppressed participants followed an immunosuppressive medication regimen or had a diagnosis of rheumatoid arthritis (*5*). For all participants, we assessed previous exposure to WNV by immunoblot for WNV envelope protein (*6*). Seroconversion to WNV was distinguished from cross-reactivity to other flaviviruses by rescreening all positive serum against a recombinant WNV-specific mutant envelope protein that lacks the conserved cross-reactive fusion loop epitope (*7*). 

We compared demographic characteristics of participant groups by using the Student *t*-test for continuous variables and χ^2^ and Fisher exact tests for categorical variables; p<0.05 indicated statistical significance. Analysis was completed with SAS software version 9.3 (SAS Institute, Cary, NC, USA) and Prism 6 (GraphPad Software, Inc., La Jolla, CA, USA).

Immunoblot detected evidence of WNV exposure in 76 (8.5%) of the 890 nonimmunosuppressed participants (Table). These seropositive participants reported neither symptoms nor diagnosis of WNV infection and are considered to have had asymptomatic infections. Timing of asymptomatic infections could not be determined, but antibodies against WNV are durable and do not differ between asymptomatic and symptomatic adults (*8*).

**Table:** Seroconversion rates among participants in study of West Nile virus, Connecticut, USA, 2000–2014*

Participant	Asymptomatic					Symptomatic, DPH case-patients 2000–2014, n = 116
Not immunosuppressed			Immunosuppressed	
Seropositive, n = 76	Seronegative, n = 814		Seropositive, n = 29	Seronegative, n = 144
Age, y, mean ± SEM	45.7 ± 2.7	48.6 ± 0.8		43.55 ± 2.5	48.48 ± 1.2	56.6 ± 1.7†
Female, %	52.6	61.1		82.8	76.4	44.0
Hispanic, %	7.9	4.1		55.2	16.7‡	Data not available
White, %	59.2	81.7§		75.9	75.0	Data not available

*DPH, Connecticut Department of Public Health. †Mean age for DPH case-patients differed statistically from that for seropositive nonimmunosuppressed (p<0.001) and seropositive immunosuppressed (p<0.001) participants. ‡Difference in percentage of Hispanics within the seropositive and seronegative categories of the immunosuppressed population (p<0.0001). §Difference in percentage of participants self-identifying as white within the seropositive and seronegative categories of the nonimmunosuppressed population (p<0.0001).

Although age is a critical risk factor for severe WNV infection (*3*,*9*), the mean age of seropositive and seronegative nonimmunosuppressed participants did not differ significantly (Table). The rate of asymptomatic seroconversion did not vary significantly among the 890 persons in 3 age groups: <35 years (42/421), 35–65 years (7/121), and >65 years (27/348) (p = 0.338). Seroconversion rates did not differ significantly by patient sex but were significantly elevated among those in self-identified Hispanic groups (p<0.0001), possibly because of different exposure histories. The similar age distribution among asymptomatic seroconverters suggests that the observed age-associated susceptibility to clinically apparent disease may result from other factors, including individual host factors and dysregulation in immune responses (*6*,*10*).

Among 173 immunosuppressed adults, 29 (16.8%) showed evidence of exposure to WNV (Table), resulting in 2.16 times the odds of positive immunoblot result than for nonimmunosuppressed adults (76/890, 8.5%; p = 0.002). Seroconversion rates among immunosuppressed persons did not differ statistically according to sex or age. The seroconversion rate was higher among immunosuppressed Hispanics (16/40, 40.0%) than non-Hispanics (13/132, 9.8%) (p<0.0001). Because the immunosuppression status of DPH-reported case-patients was not available, we could not further explore a role for immunosuppression in the occurrence of WNV infection among these patients. Immunosuppression carries unique risks for infectious diseases; thus, the higher rate of seroconversion among immunosuppressed participants may be a consequence of underlying medical conditions or medication regimens.

The mean age for asymptomatic seropositive adults, nonimmunosuppressed and immunosuppressed, was lower than that for DPH-reported symptomatic case-patients (Table; p = 0.0004). The 2 groups did not vary significantly according to sex (p = 0.30). Because racial data for DPH-reported case-patients was not available, no comparison by race was possible. Comparison of geocoded household locations of all study participants and DPH case-patients showed an overlapping distribution of nonimmunosuppressed and immunosuppressed asymptomatic seroconverters and DPH case-patients (Technical Appendix Figure). Although only a surrogate for location where infection was acquired, this mapping provides no support for localized pockets of increased disease susceptibility.

We provide evidence of WNV exposure in Connecticut among 1,063 adults who differed by age, sex, race, and immunosuppression status. Among nonimmunosuppressed asymptomatic participants, age was not a significant factor with regard to WNV seroconversion. However, mean age of symptomatic case-patients was older than that of asymptomatic seropositive participants, indicating that age remains a factor in disease susceptibility (*9*). Age has a well-documented role in decreased immune cell function and increased susceptibility to infectious diseases, including WNV (*9*,*10*); dysregulation of immune responses with elevated cytokine levels may contribute to development of severe disease. The higher WNV seroprevalence among immunosuppressed adults strongly suggests a key role for immune factors in seroconversion.

Ongoing research seeks to further define the immune system attributes that lead to increased risk for higher WNV disease severity; active areas of interest include genomic, transcriptional, and immune- and age-related variable responses (*6*,*8*,*9*). In addition to environmental conditions that affect vector abundance, our study suggests that individual variation, such as immune status, may be a key driver for susceptibility to infection and disease severity and for differing seroconversion rates among neighbors.

Technical Appendix Geocoded locations of West Nile virus study participants throughout Connecticut, USA, 2000–2014.
