# Supplementary material for: West Nile Virus Seroprevalence, Connecticut, USA, 2000–2014
Source: Emerg Infect Dis. 2017 Apr;23(4):708–10. doi: 10.3201/eid2304.161669 (PMC5367428; doi:10.3201/eid2304.161669)
Supplement: Technical Appendix — Geocoded locations of West Nile virus study participants throughout Connecticut, USA, 2000–2014. [file 16-1669-Techapp-s1.pdf]

Article DOI: http://dx.doi.org/10.3201/eid2304.161669

West Nile Virus Seroprevalence, Connecticut, USA, 2000–2014

Technical Appendix

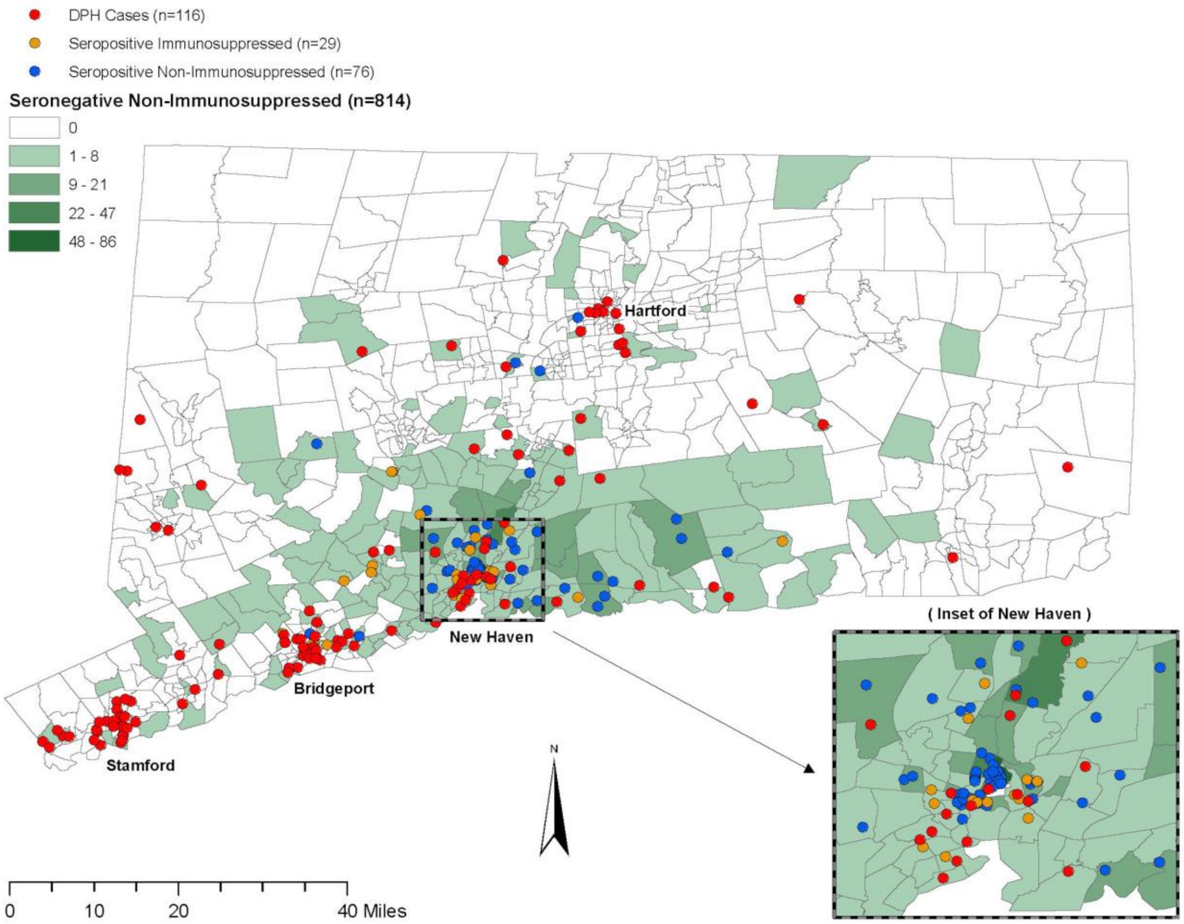

Technical Appendix Figure. Geocoded locations of West Nile Virus study subjects throughout the state of Connecticut, USA. Dots depict the geocoded household location of seronegative and asymptomatic seropositive subjects (non-immunosuppressed subjects, blue dots; immunosuppressed subjects, orange dots) (Table) and DPH cases (red dots) using spatial skewing to preserve protected health information. The home residence at the time of study enrollment was used as a surrogate for exposure location for all subjects (ArcGIS software; ArcMap 10.3, Esri, Redlands, California USA). Green shading indicates density of study enrollment by census tract. Inset box of the city of New Haven shows overlapping of DPH

cases, asymptomatic seropositive subjects, and seronegative subjects, suggesting similar exposure to WNV-infected mosquitoes.
